# Supplementary material for: Host diet and evolutionary history explain different aspects of gut microbiome diversity among vertebrate clades
Source: Nat Commun. 2019 May 16;10:2200. doi: 10.1038/s41467-019-10191-3 (PMC6522487; doi:10.1038/s41467-019-10191-3)
Supplement: Supplementary file 1 — Supplementary Information [file 41467_2019_10191_MOESM1_ESM.pdf]

Supplementary Information

Host diet and evolutionary history explain different aspects of gut microbiome diversity among vertebrate clades

Youngblut, Reischer et al.

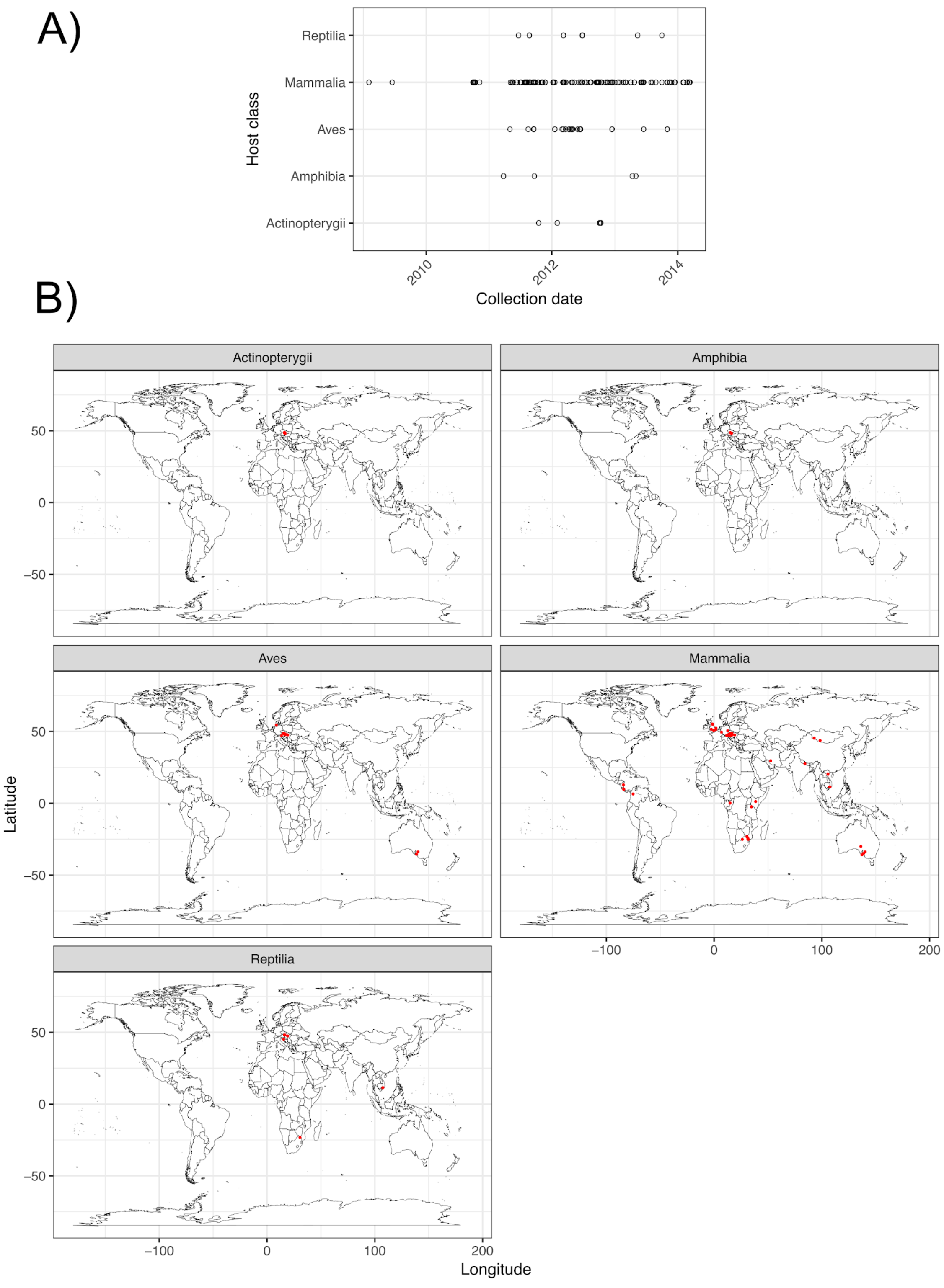

Supplementary Figure 1. The dataset includes gut microbiome samples spanning 5 host classes, ~5 years, and 6 continents. A) the time point of sample collection B) the geographic location of each sample collection. Source data are provided as a Source Data file.

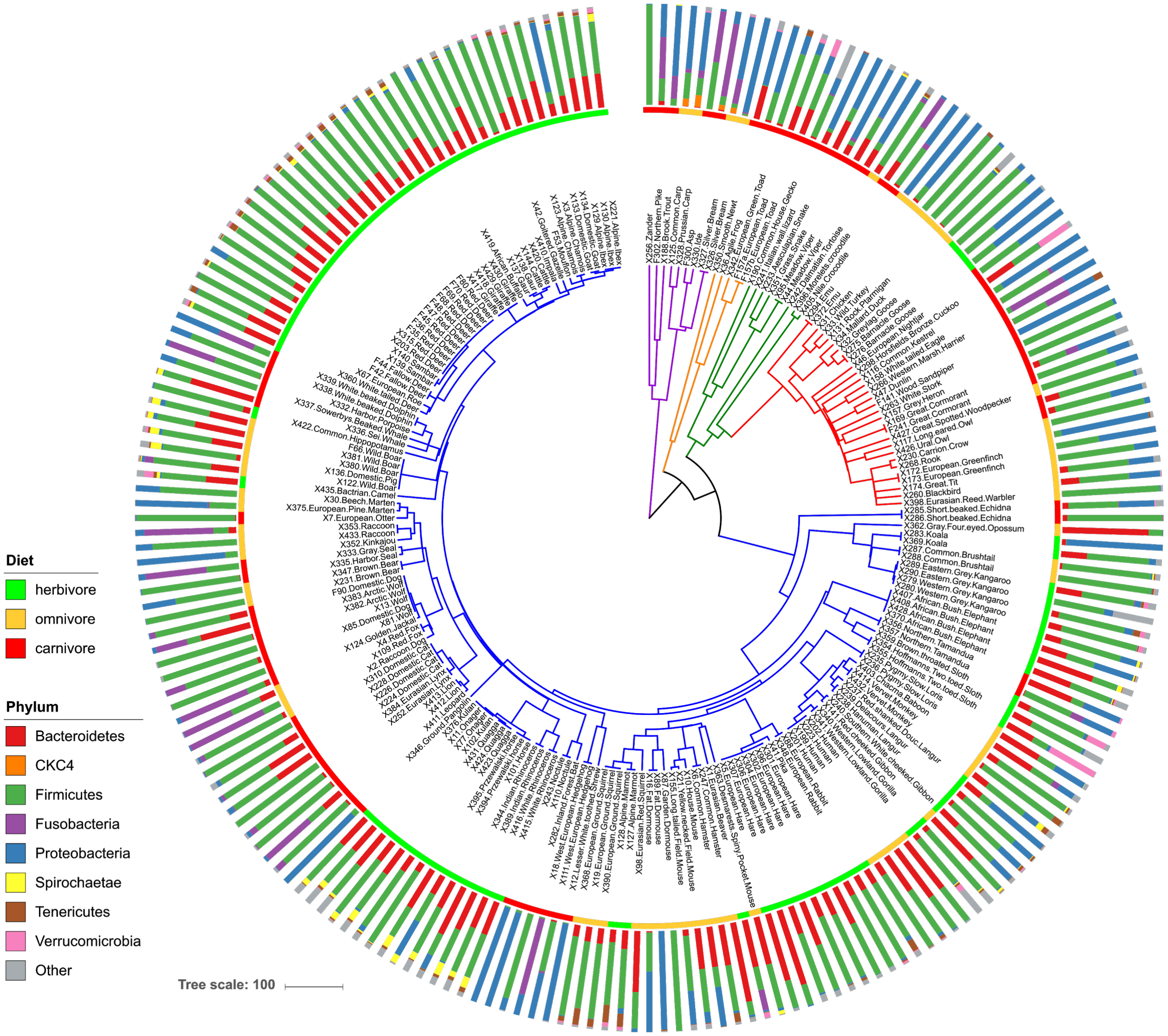

Supplementary Figure 2. Phylum-level grouping of microbiome diversity by host phylogeny and host metadata. The host phylogeny is that same as shown in Fig. 1, except tips have been expanded to include all individuals of each species. Host diet and relative abundances of microbial phyla are mapped onto the tree. Source data are provided as a Source Data file.

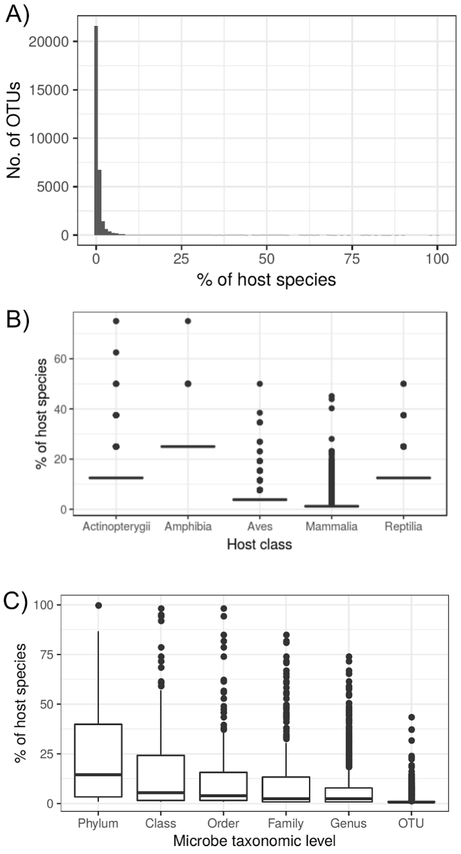

D)

Taxonomic level	Group	Prevalence
Phylum	Firmicutes	100
Phylum	Proteobacteria	100
Class	Firmicutes;Clostridia	98.4
Order	Firmicutes;Clostridia;Clostridiales	98.4
Class	Proteobacteria;Betaproteobacteria	95.3
Class	Proteobacteria;Gammaproteobacteria	94.5
Order	Proteobacteria;Betaproteobacteria;Burkholderiales	94.5
Class	Firmicutes;Bacilli	92.2
Phylum	Actinobacteria	86.7
Phylum	Bacteroidetes	85.9
Order	Proteobacteria;Gammaproteobacteria;Enterobacteriales	85.2
Family	Proteobacteria;Gammaproteobacteria;Enterobacteriales; Enterobacteriaceae	85.2
Order	Firmicutes;Bacilli;Lactobacillales	82
Family	Firmicutes;Clostridia;Clostridiales;Lachnospiraceae	82
Family	Firmicutes;Clostridia;Clostridiales;Clostridiaceae 1	81.2
Class	Bacteroidetes;Bacteroidia	78.9
Order	Bacteroidetes;Bacteroidia;Bacteroidales	78.9

Supplementary Figure 3. OTUs are sparsely distributed in the dataset. A) OTU prevalence across all host species (found in at least one individual). B) OTU prevalence across host species, grouped by host class. C) Microbe taxonomic group prevalence across all host species. D) Microbe taxonomic groups with a prevalence of >75 % across all host species. Box centerlines, edges, whiskers, and points signify the median, IQR, 1.5 * IQR, and >1.5 * IQR, respectively. Source data are provided as a Source Data file.

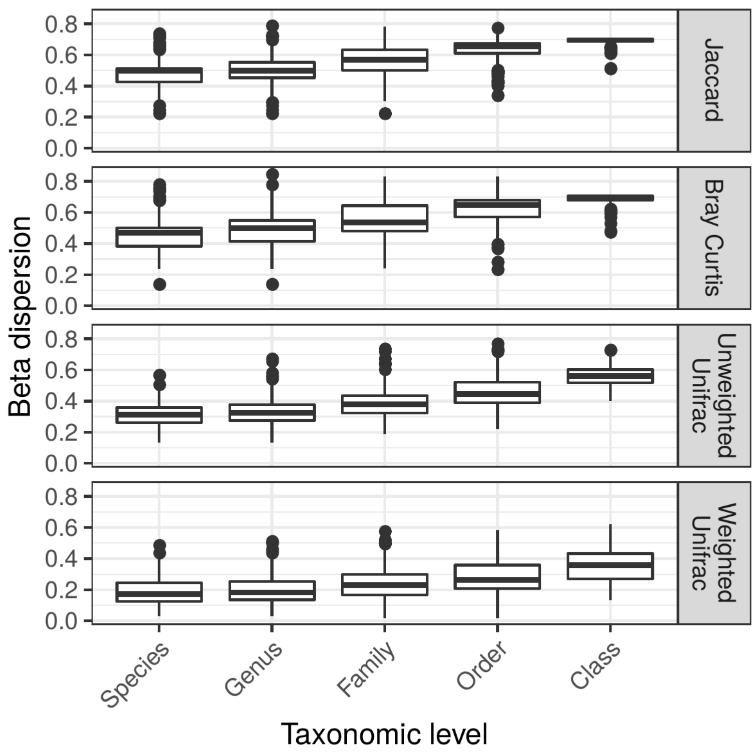

Supplementary Figure 4. Beta-diversity is more constrained at finer taxonomic levels. The boxplots show the distribution of beta-dispersion (distance from multivariate centroids of the group), with groups defined as host clades at each taxonomic level. Box centerlines, edges, whiskers, and points signify the median, IQR, 1.5 * IQR, and >1.5 * IQR, respectively. Source data are provided as a Source Data file.

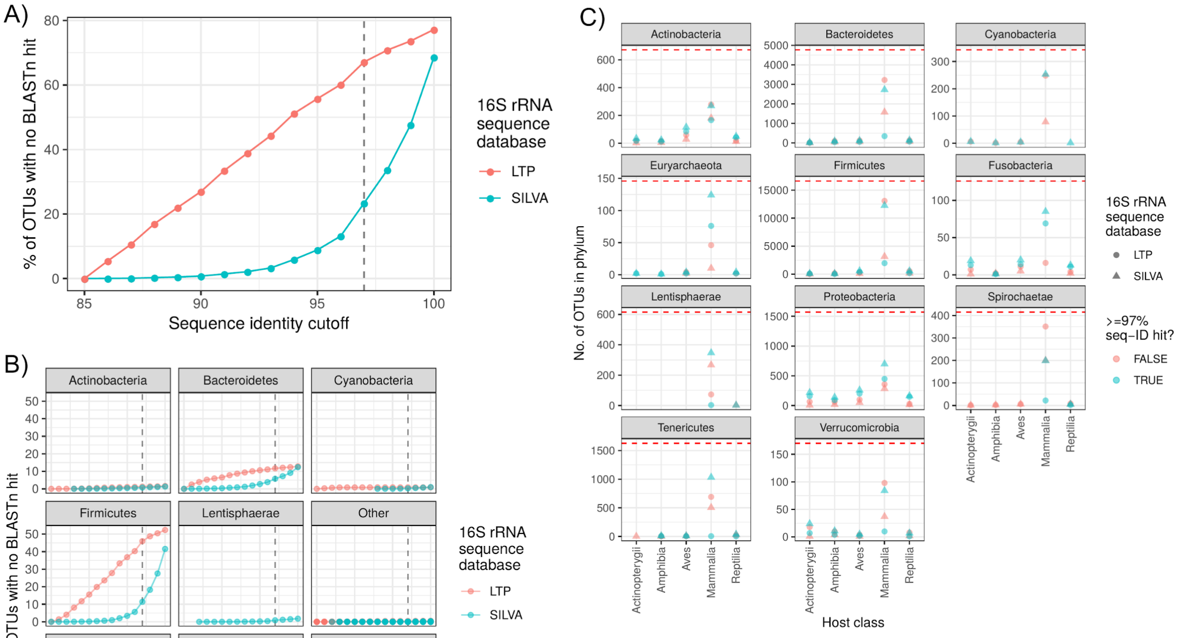

Supplementary Figure 5. Many of the OTUs in the dataset lack (un)cultured representatives. The plots show the distribution of BLASTn hits between OTU representative sequences and 16S rRNA sequences of cultured representative taxa in the SILVA All Species Living Tree database ("LTP") or the entire SILVA database de-replicated at 99% sequence identity ("SILVA"). The vertical dashed line in A) and B) signifies a percent sequence identity of 97 %. "Other" in B) comprises all other phyla. ">=97% seq-ID hit?" in C) signifies whether the OTU has at least one BLASTn hit to a taxon in either SILVA database with ≥97 % sequence identity. For each category in C), only OTUs with a prevalence of >0 are counted, while the dashed line signifies all OTUs in that phylum. Only phyla with >100 OTUs are shown in C). Source data are provided as a Source Data file.

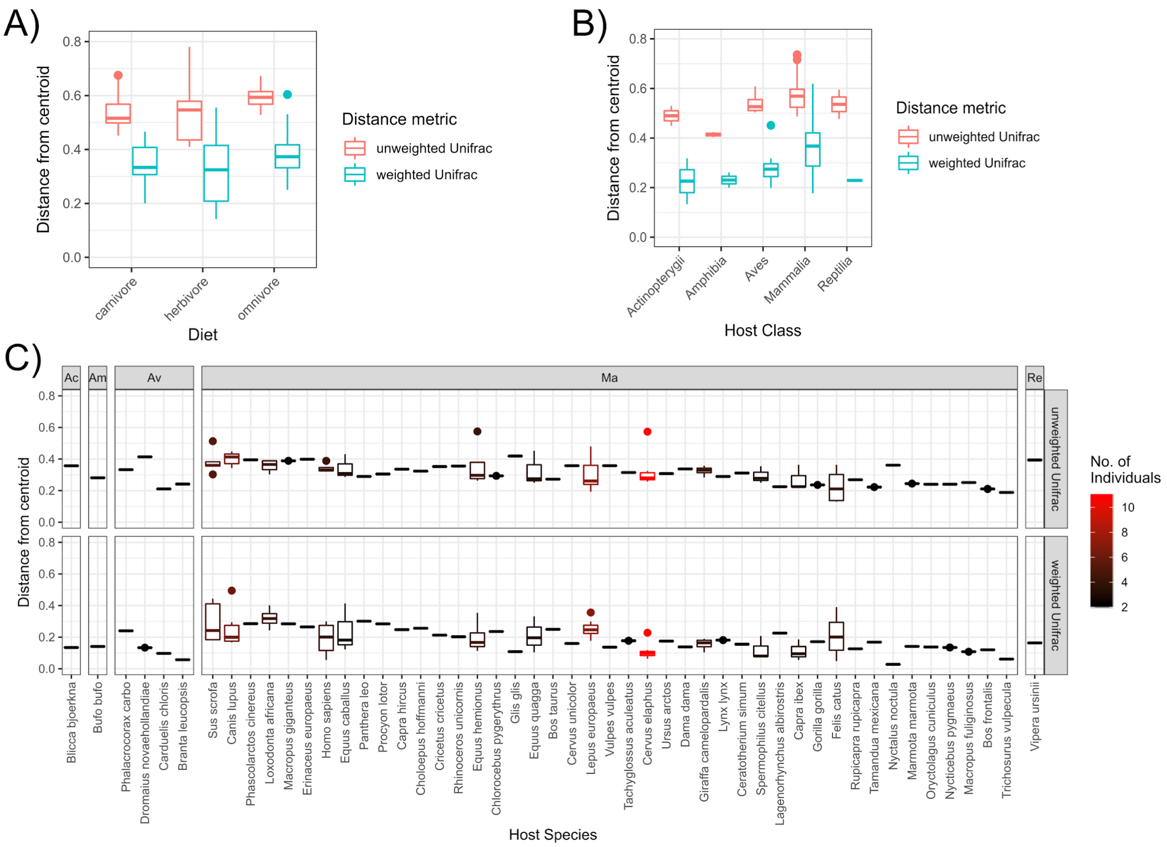

Supplementary Figure 6. Less multivariate dispersion for weighted versus unweighted Unifrac values. The boxplots show the distances from multivariate centroides (PCA of weighted or unweighted Unifrac values) for samples grouped by A) host diet B) host taxonomy C) host species. The plots in C) are x-faceted by host class ("Ac" = Actinopterygii, "Am" = Amphibia, "Av" = Aves, "Ma" = Mammalia, "Re" = Reptilia), and the boxplots are colored by the number of individuals per species. Only species with ≥2 samples (No. of samples = 135; No. of species = 50) are shown in C). Box centerlines, edges, whiskers, and points signify the median, IQR, 1.5 * IQR, and >1.5 * IQR, respectively. Source data are provided as a Source Data file.

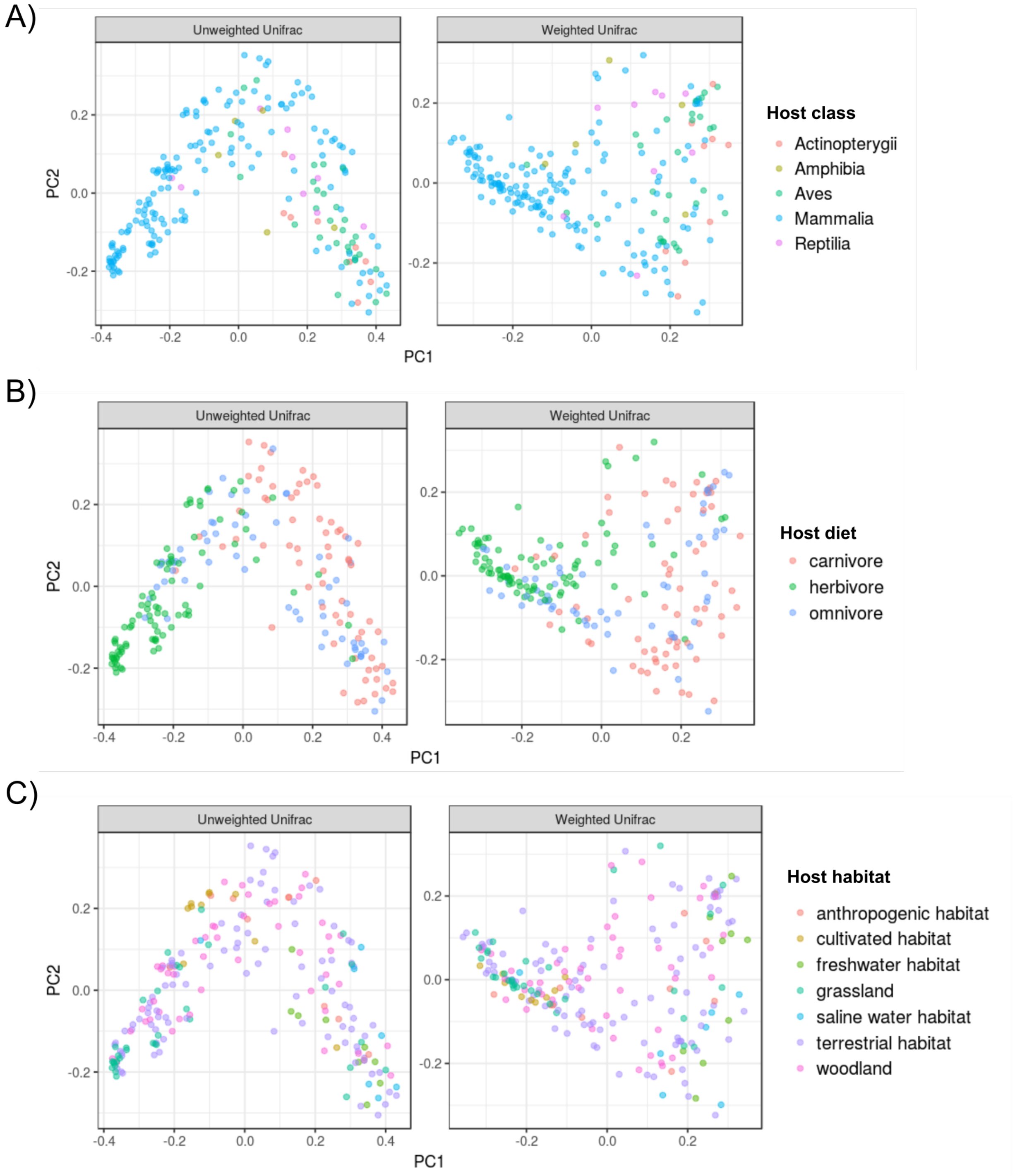

Supplementary Figure 7. PCoA plots of microbiome beta-diversity reveal some grouping by host taxonomy and diet. Principal coordinates (PCoA) ordinations of unweighted and weighted Unifrac distances among all samples, with samples colored by host A) class B) diet C) habitat. The variance explained by PC1 and PC2 of the unweighted Unifrac PCoA is 17 and 6 %, respectively. The variance explained by PC1 and PC2 of the weighted Unifrac PCoA is 26 and 11 %, respectively. Source data are provided as a Source Data file.

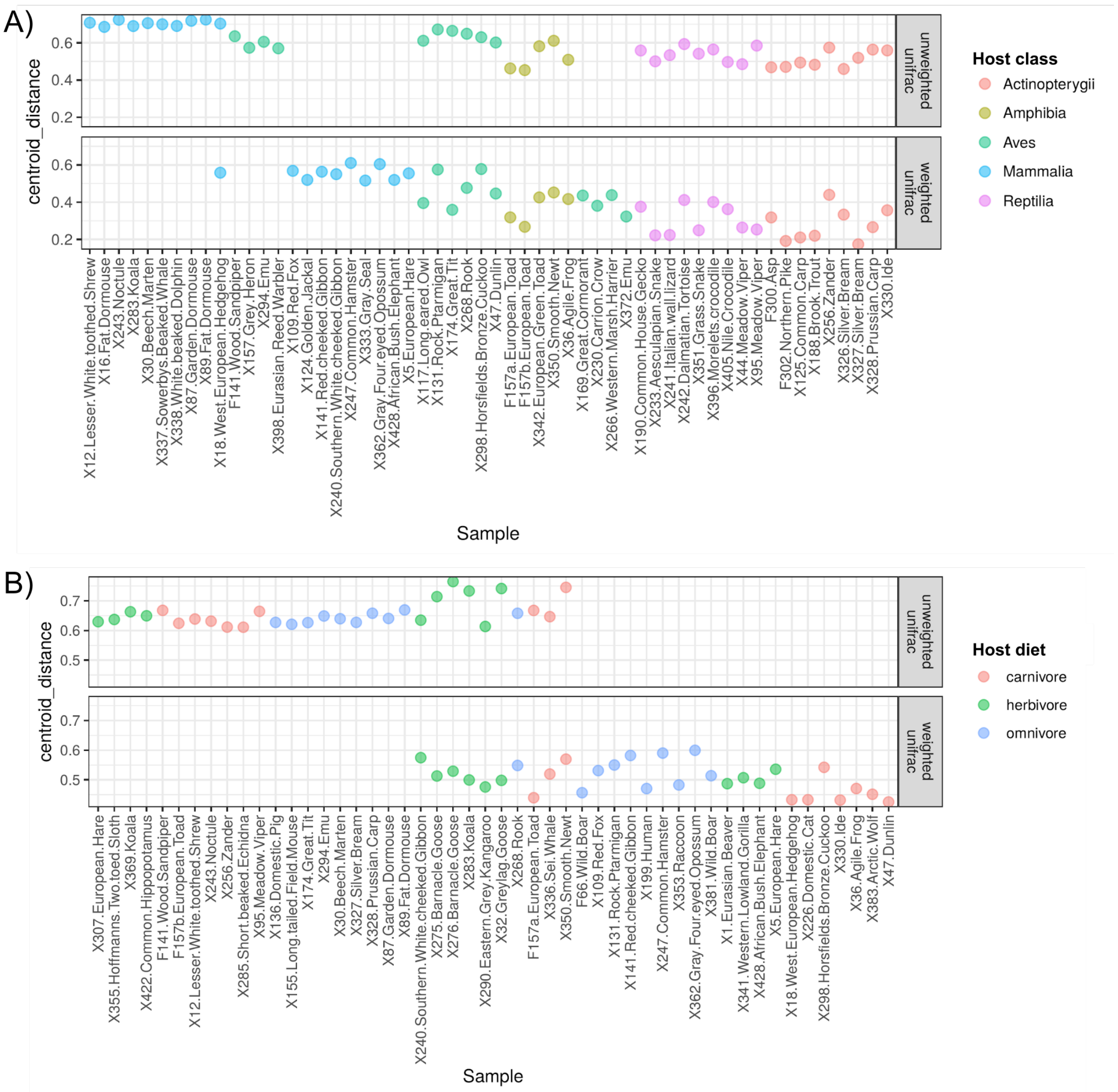

Supplementary Figure 8. Outlier samples differ among beta-diversity metrics and groupings. Centroid distance of samples for unweighted or weighted Unifrac, with centroids defined by host taxonomy (A) or host diet (B). Only samples with a centroid distance of >0.4 (i.e., the largest outliers) are shown. Source data are provided as a Source Data file.

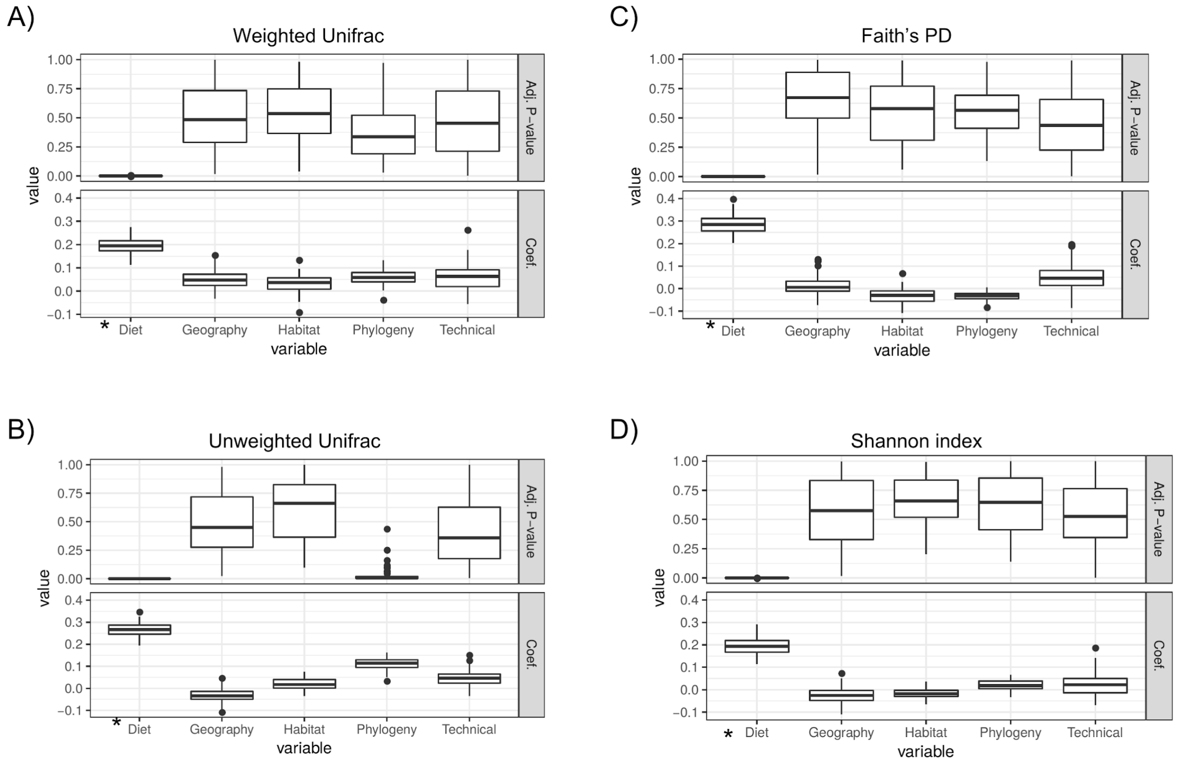

Supplementary Figure 9. Very similar MRM results obtained if selecting just one sample per family, which reduces sample size biases towards Mammalia. The figure is the same as Fig. 2, but for each dataset subset, only one sample was selected per family instead of per species. "*" denotes significance (Adj. $p < 0.05$ for ≥95 % of dataset subsets; see Methods). Box centerlines, edges, whiskers, and points signify the median, IQR, 1.5 * IQR, and >1.5 * IQR, respectively. Source data are provided as a Source Data file.

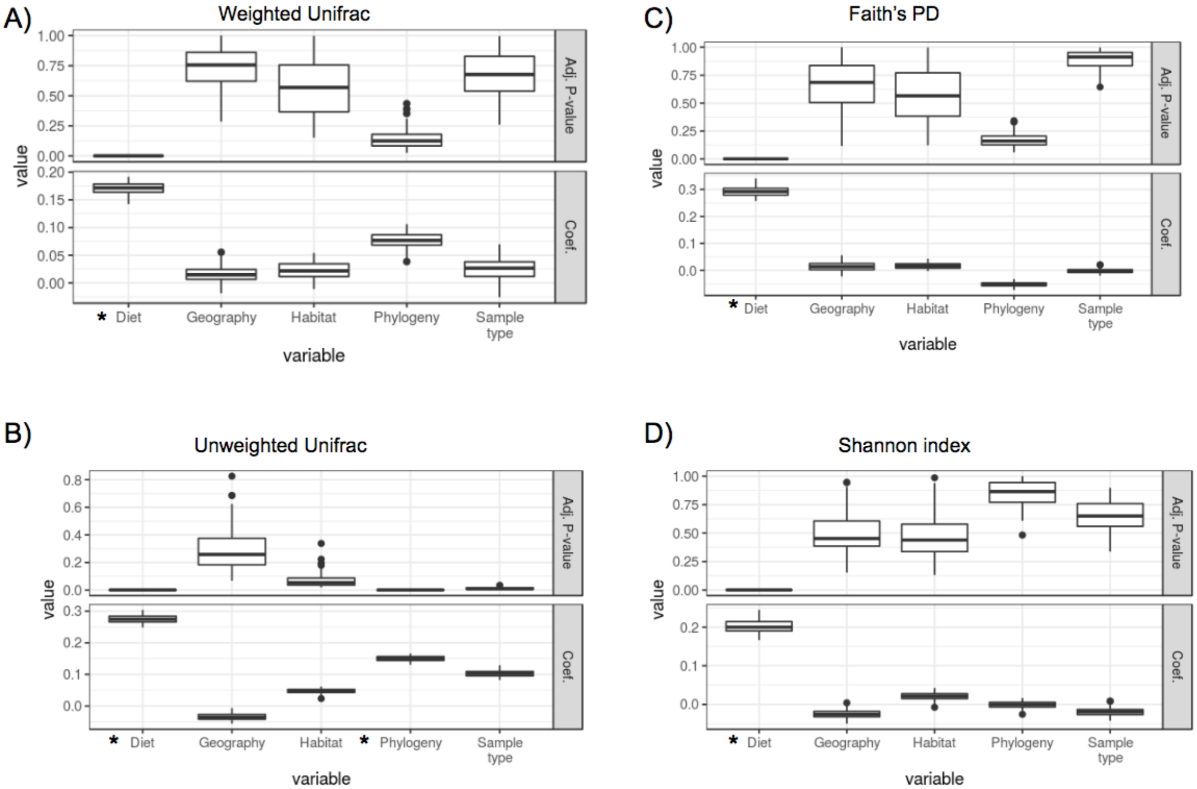

Supplementary Figure 10. The same analysis as in Fig. 2, but only wild hosts were included (total samples = 170; total host species = 119). “*” denotes significance (Adj. $p < 0.05$ for ≥95 % of dataset subsets; see Methods). Box centerlines, edges, whiskers, and points signify the median, IQR, 1.5 * IQR, and >1.5 * IQR, respectively. Source data are provided as a Source Data file.

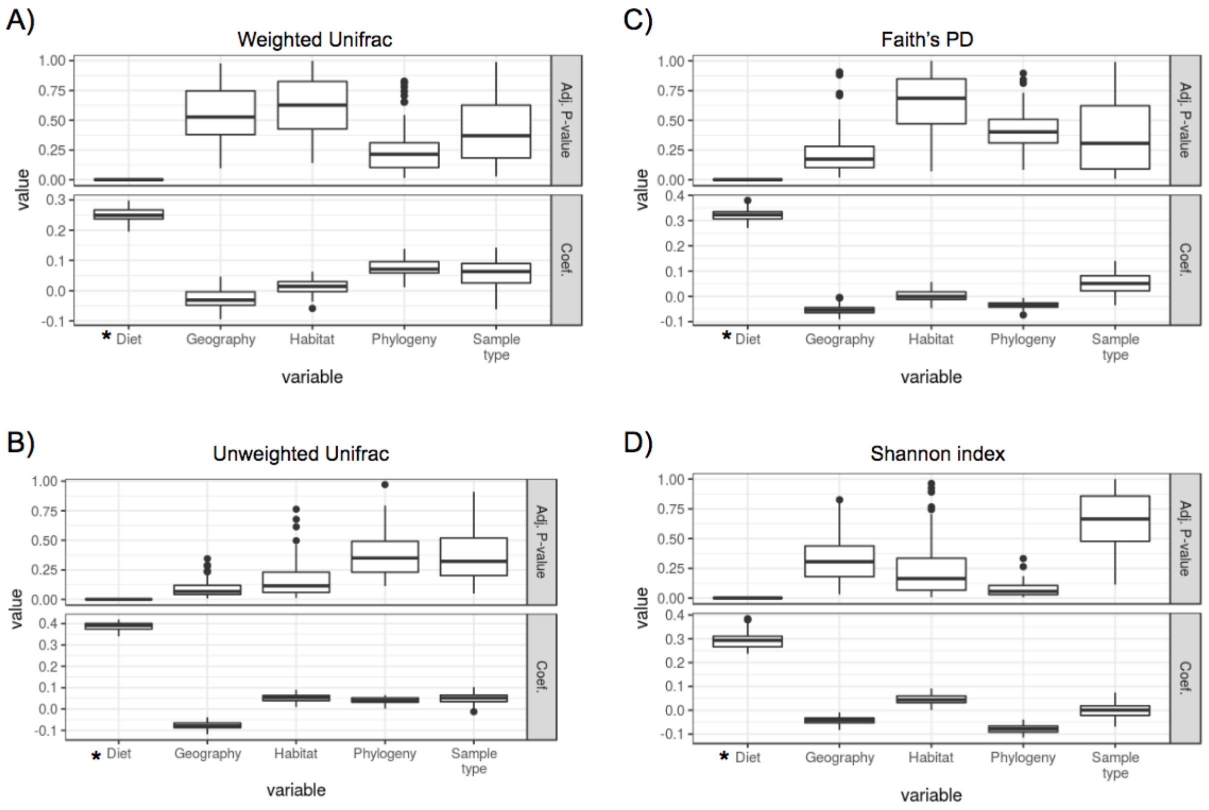

Supplementary Figure 11. The same analysis as in Fig. 2, but only mammalian hosts were included (total samples = 160; total host species = 82). "*" denotes significance (Adj. $p < 0.05$ for ≥95 % of dataset subsets; see Methods). Box centerlines, edges, whiskers, and points signify the median, IQR, 1.5 * IQR, and >1.5 * IQR, respectively. Source data are provided as a Source Data file.

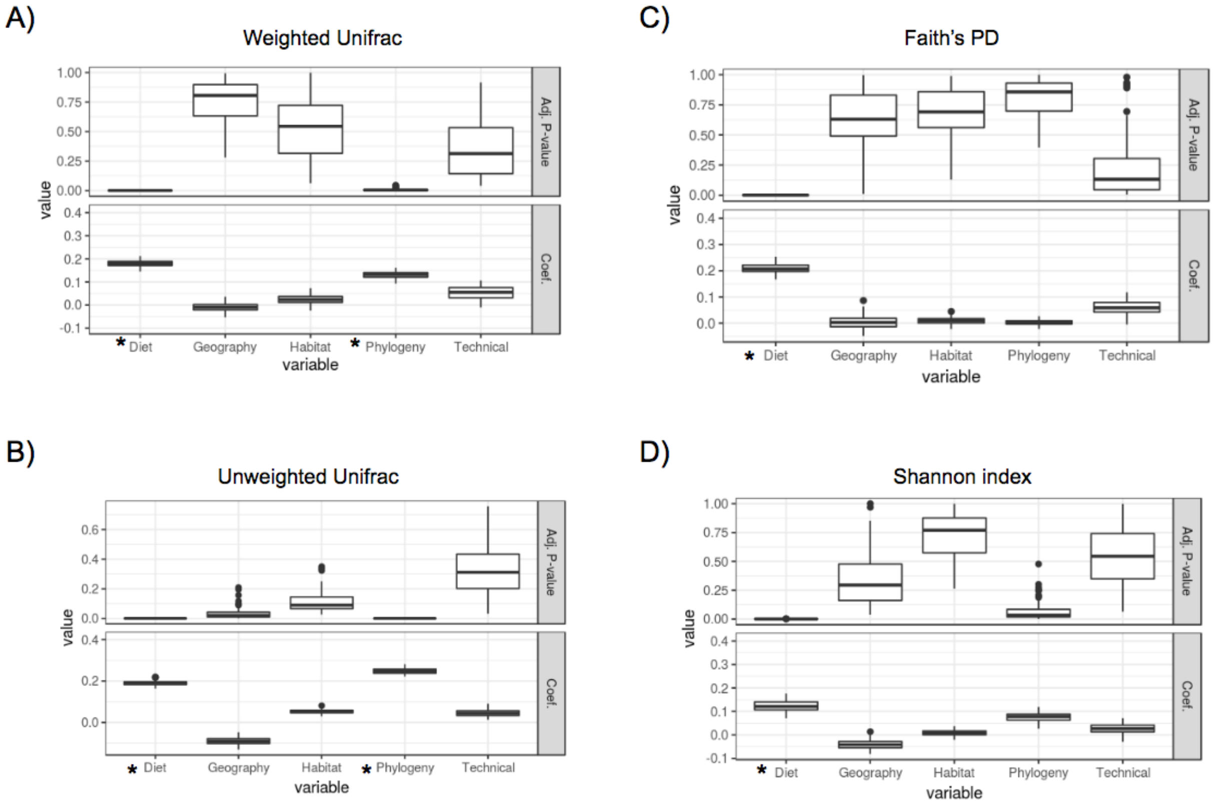

Supplementary Figure 12. The same analysis as in Fig. 2, but OTUs were first aggregated at the genus level. "*" denotes significance (Adj. $p < 0.05$ for ≥95 % of dataset subsets; see Methods). Box centerlines, edges, whiskers, and points signify the median, IQR, 1.5 * IQR, and >1.5 * IQR, respectively. Source data are provided as a Source Data file.

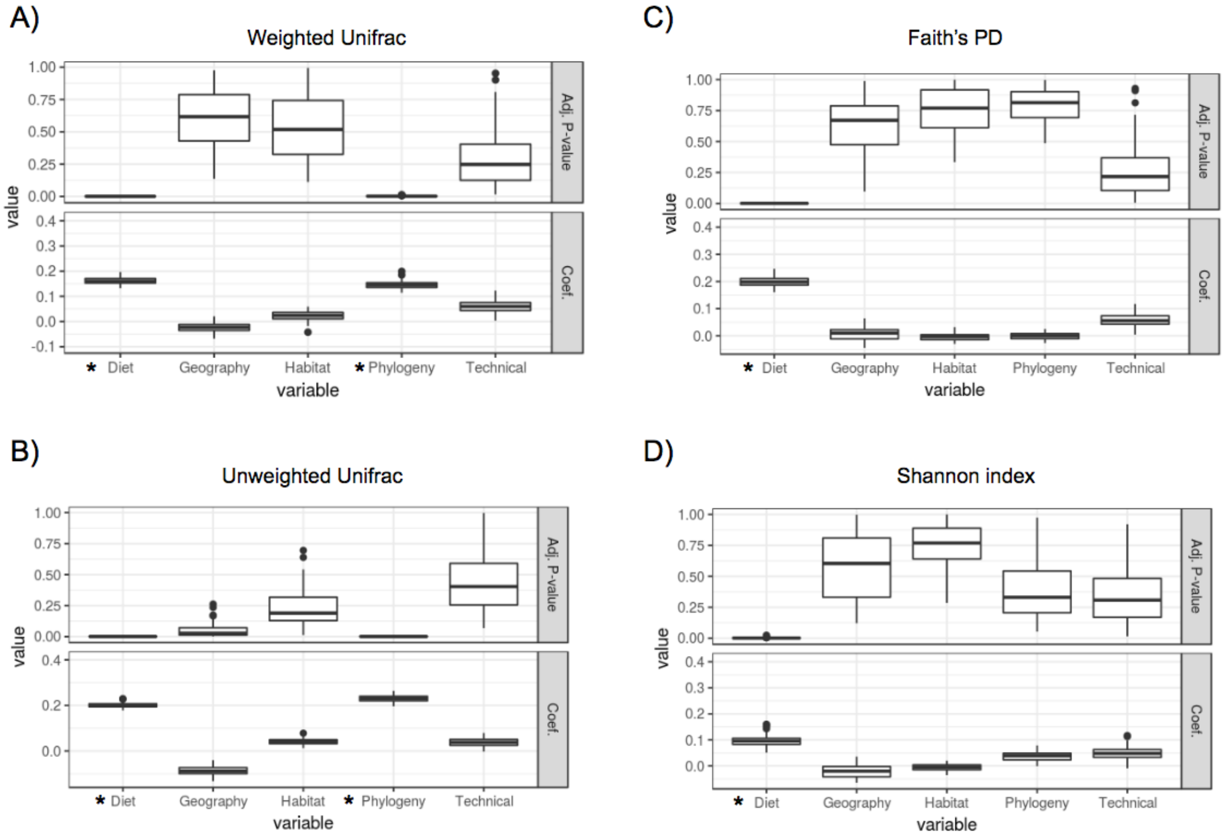

Supplementary Figure 13. The same analysis as in Fig. 2, but OTUs were first aggregated at the family level. “*” denotes significance (Adj. p < 0.05 for ≥95 % of dataset subsets; see Methods). Box centerlines, edges, whiskers, and points signify the median, IQR, 1.5 * IQR, and >1.5 * IQR, respectively. Source data are provided as a Source Data file.

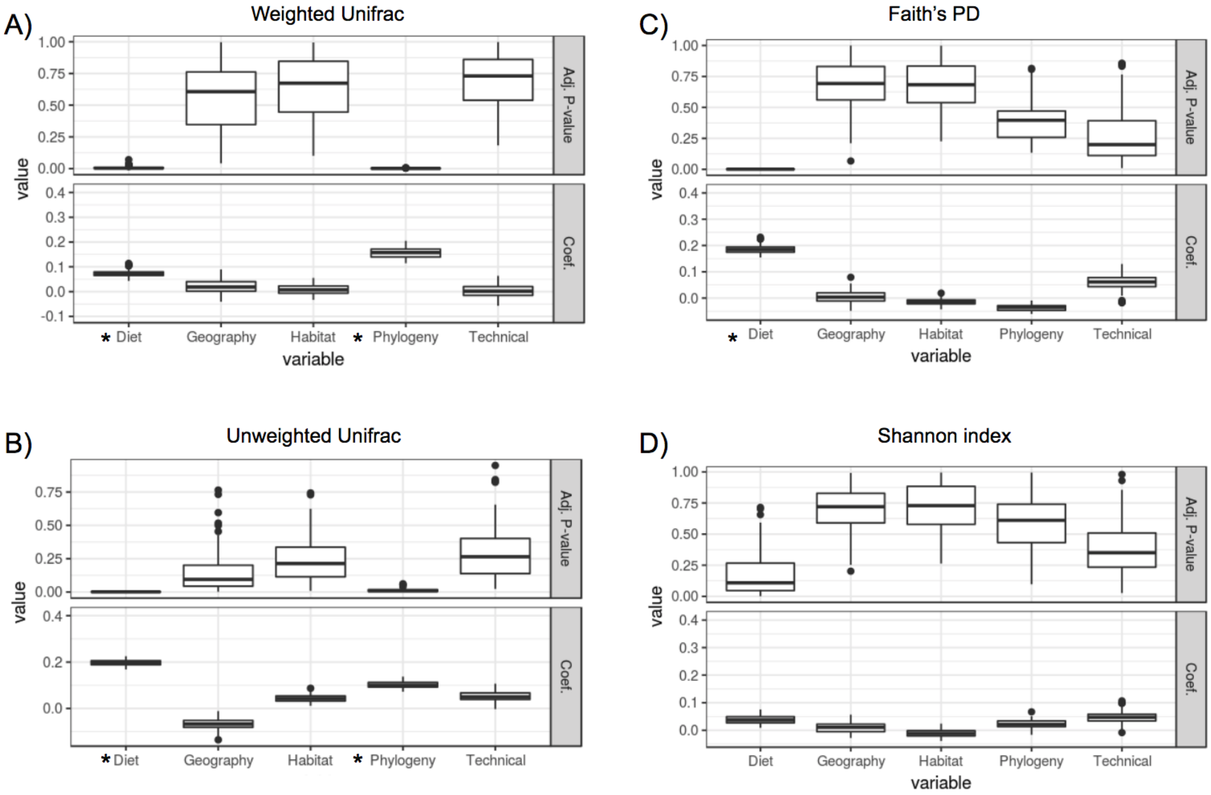

Supplementary Figure 14. The same analysis as in Fig. 2, but OTUs were first aggregated at the class level. "*" denotes significance (Adj. $p < 0.05$ for ≥95 % of dataset subsets; see Methods). Box centerlines, edges, whiskers, and points signify the median, IQR, 1.5 * IQR, and >1.5 * IQR, respectively. Source data are provided as a Source Data file.

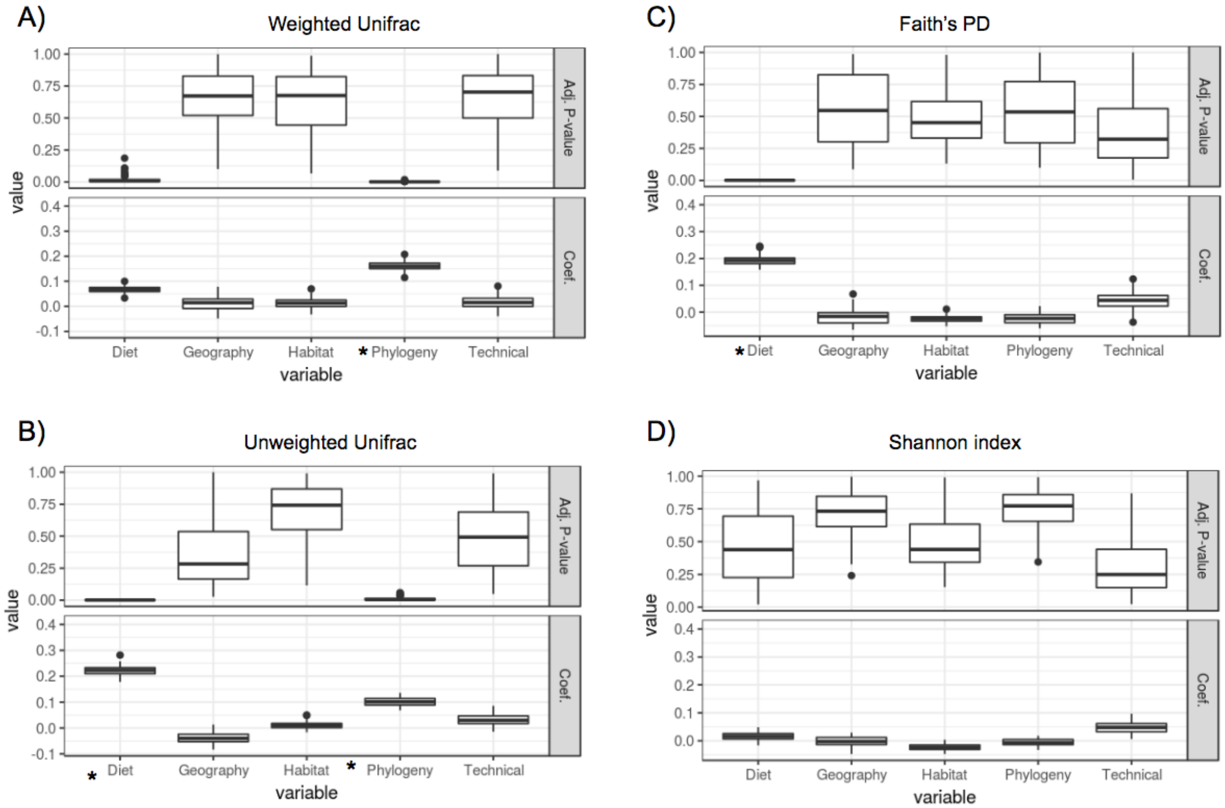

Supplementary Figure 15. The same analysis as in Fig. 2, but OTUs were first aggregated at the phylum level. “*” denotes significance (Adj. $p < 0.05$ for ≥95 % of dataset subsets; see Methods). Box centerlines, edges, whiskers, and points signify the median, IQR, 1.5 * IQR, and >1.5 * IQR, respectively. Source data are provided as a Source Data file.

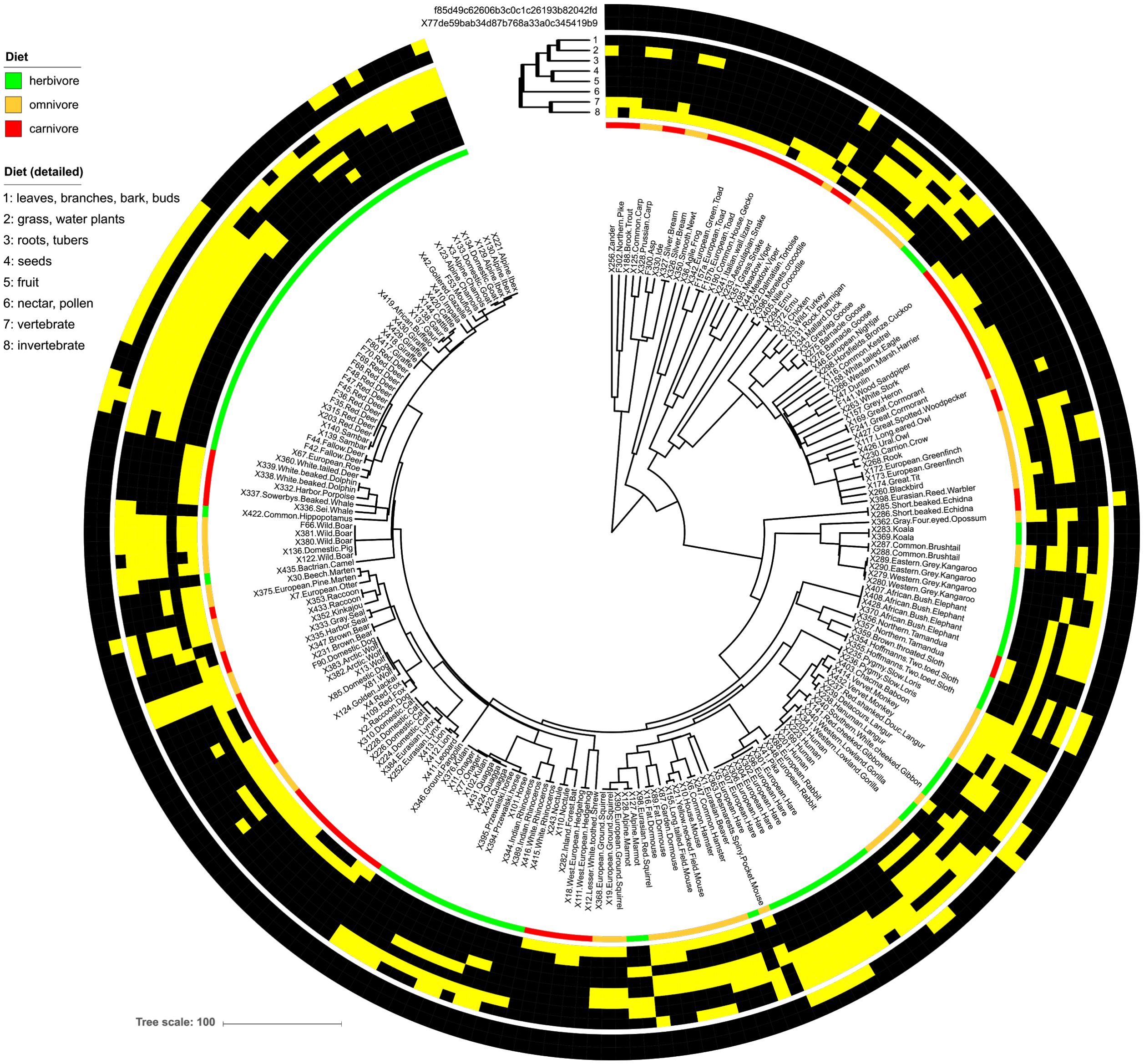

Supplementary Figure 16. OTUs significantly explained by diet differ in their prevalence among hosts and diets. The phylogeny is the same as shown in Supplementary Fig. 2. The presence of the PGLS-significant OTUs (See Fig. 3) are mapped onto the host tree (outer ring) along with the detailed host diet characteristics (middle ring) and the general diet (inner ring). Yellow and black squares signify presence and absence, respectively. Source data are provided as a Source Data file.

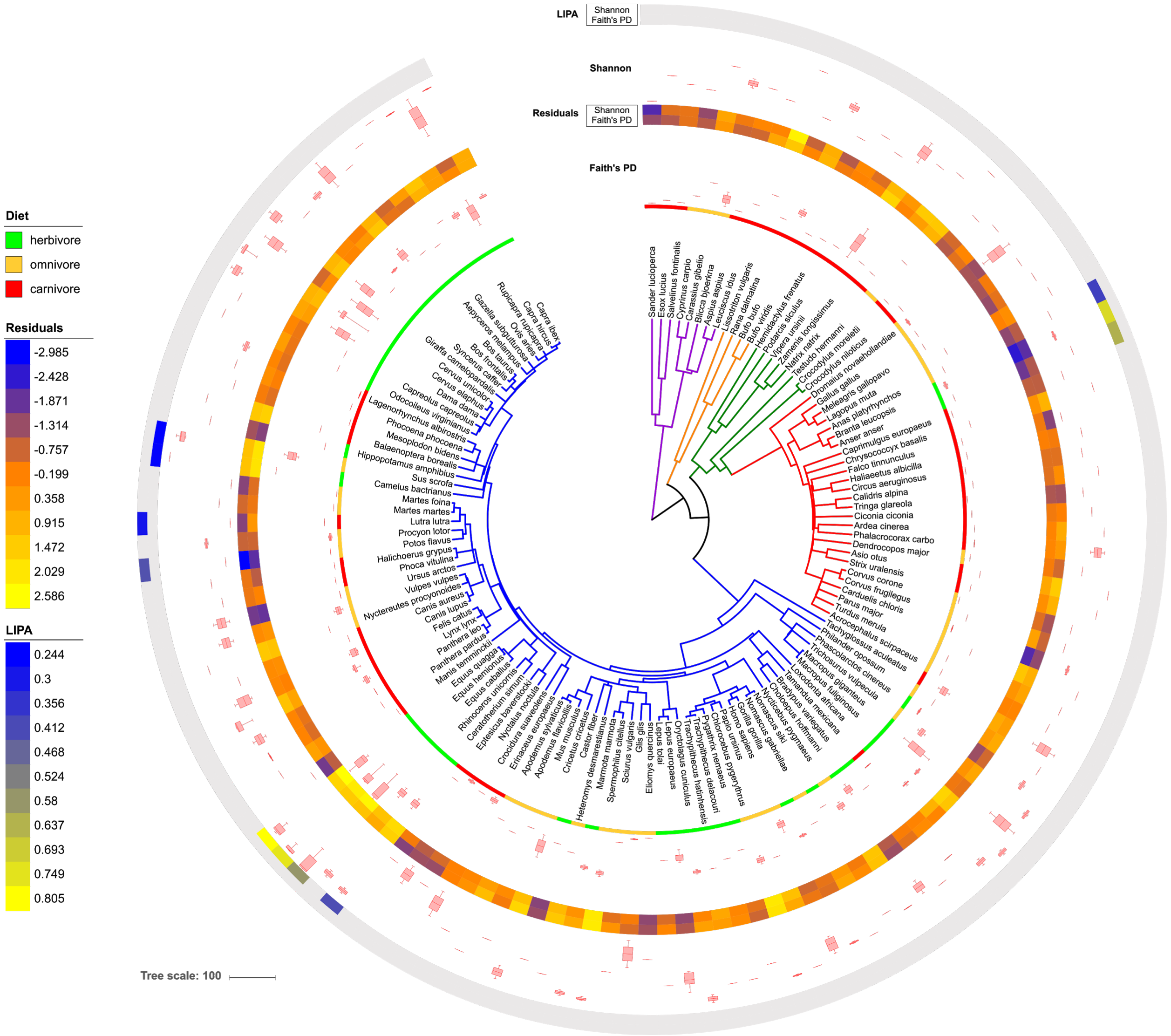

Supplementary Figure 17. **Very little phylogenetic signal of alpha-diversity after accounting for diet.** The phylogeny is the same as shown in Fig. 1. The boxplots show distributions of alpha-diversity values (inner = Faith's PD; outer = Shannon index) among samples for the same host species. The heatmap between the boxplots shows residuals for both alpha-diversity measures after regressing out diet (all diet components). The outer heatmap shows LIPA Moran's I index values (i.e., local phylogenetic signal), with grey representing all non-significant (significant defined as Adj. $p < 0.05$). Box centerlines, edges, and whiskers signify the median, IQR, 1.5 * IQR. Source data are provided as a Source Data file.

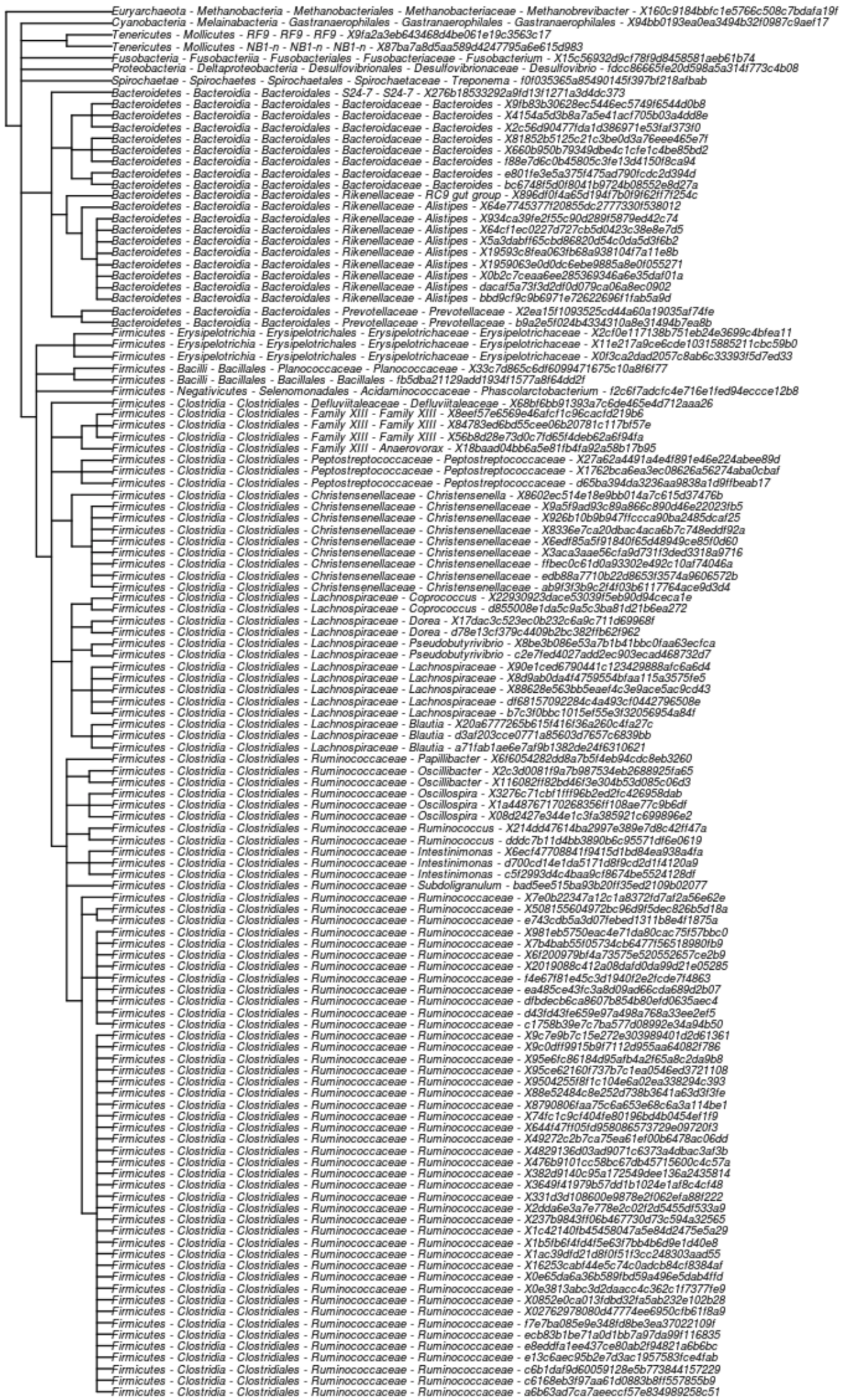

Supplementary Figure 18. The cladogram is the same as in Fig. 4, but tip labels show the full taxonomy of each OTU: “phylum - class - order - family - genus - OTU-ID”.

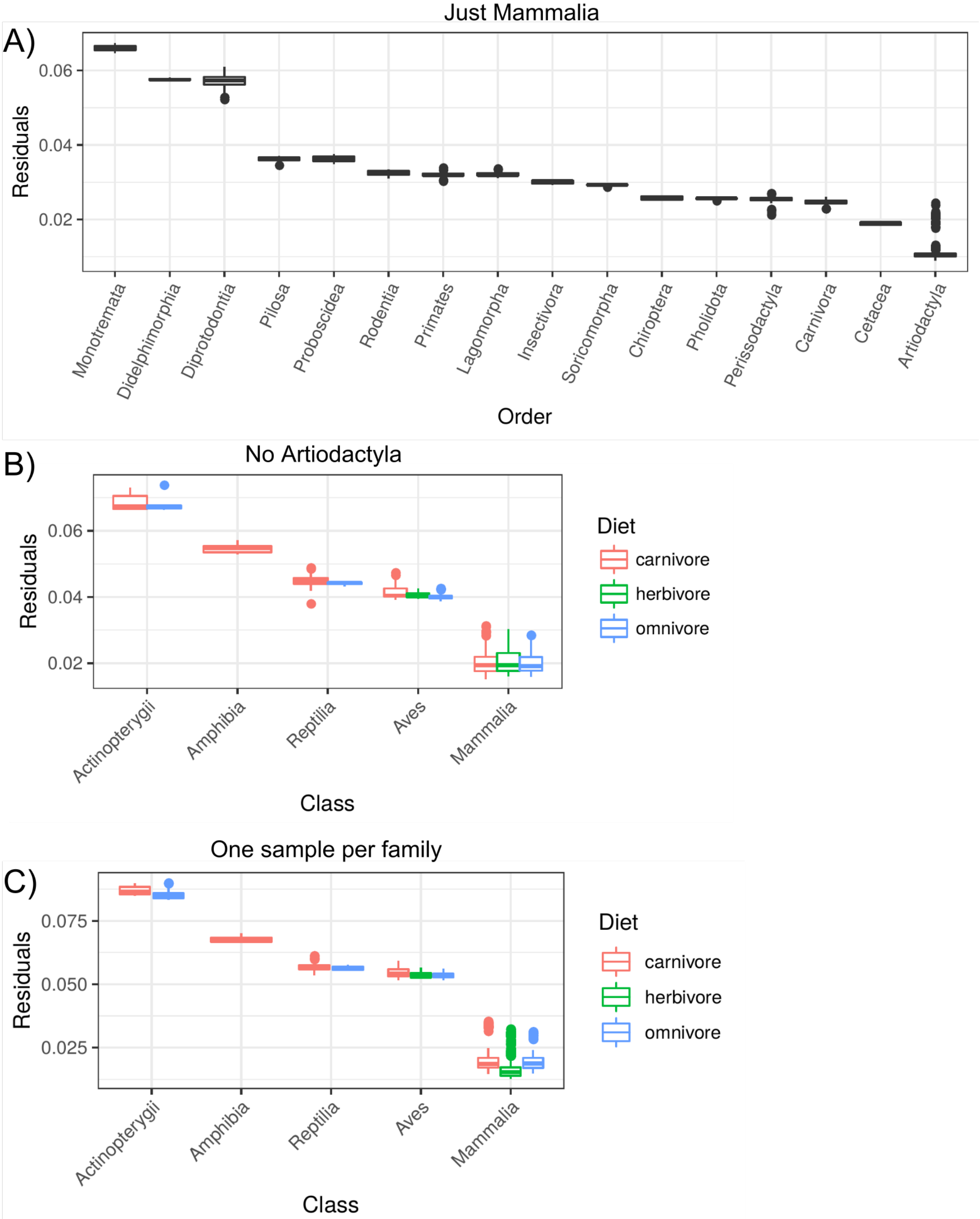

Supplementary Figure 19. The PACo test of co-phylogeny is robust to removing portions of the dataset. The boxplots show Procrustean residuals for each host clade (smaller residuals means a better fit). PACo was performed on dataset subsets consisting of either A) just Mammalia hosts, B) Artiodactyla host removed, or C) just one sample per host family used. Box centerlines, edges, whiskers, and points signify the median, IQR, 1.5 * IQR, and >1.5 * IQR, respectively. Source data are provided as a Source Data file.

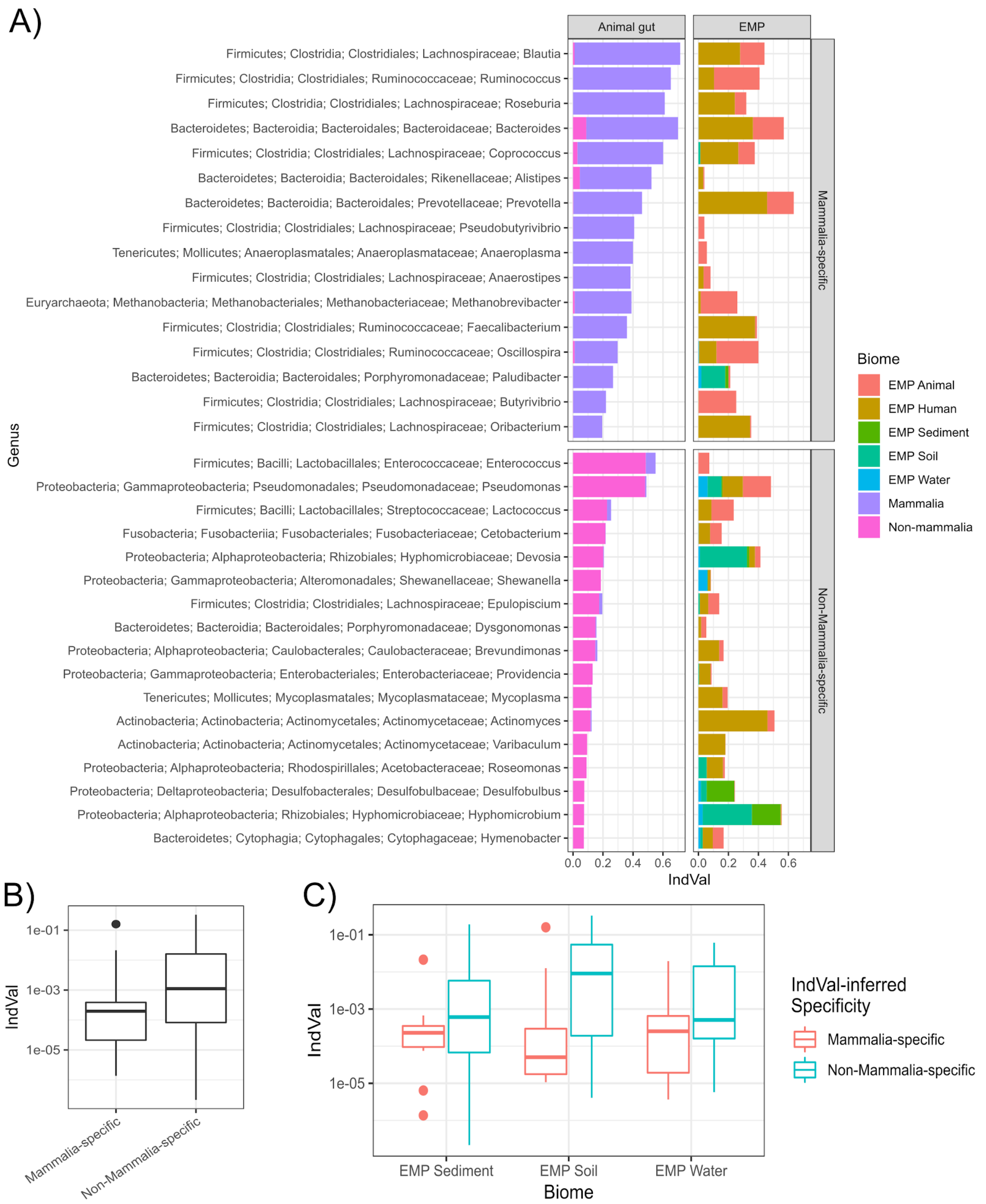

Supplementary Figure 20. Evidence that taxa specific to non-mammal hosts are distributed more in non-animal/human biomes. A) The bar plots show Indicator Values ("IndVal") for biome specificity in either the animal gut microbiome dataset of this study ("Animal gut") or the Earth Microbiome Project ("EMP"). OTUs abundances among samples in each biome from each dataset were summed at the genus level. Only genera overlapping among datasets and having a BH-adjusted IndVal p-value of <0.05 are included. For the EMP dataset, biome IDs were manually assigned (No. of samples: Animal = 317, Human = 206, Sediment = 259, Soil = 193, Water = 242). The boxplots summarize the IndVal effect sizes for each mammal-specific and non-mammal-specific genus (determined by IndVal significance) in B) all environmental biome or C) each environmental biome. Box centerlines, edges, whiskers, and points signify the median, IQR, 1.5 * IQR, and >1.5 * IQR, respectively. Source data are provided as a Source Data file.

A)

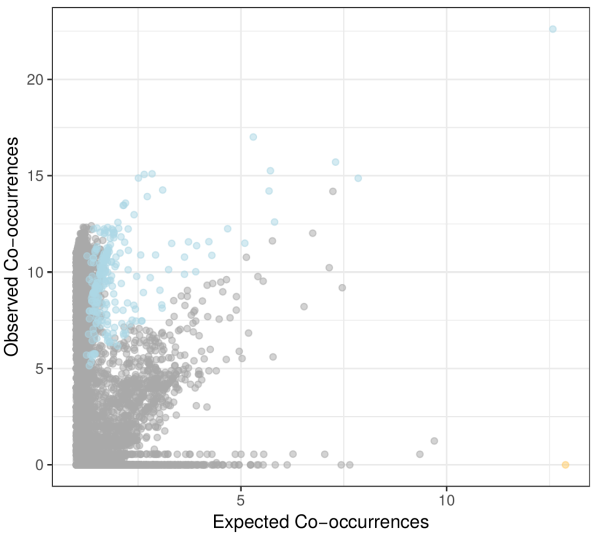

B)

Phylum (x)	Phylum (y)	Sign	Perc. of Edges	Edges Norm.
Proteobacteria	Proteobacteria	-1	0.84	0.024
Proteobacteria	Proteobacteria	1	5.88	0.167
Proteobacteria	Firmicutes	1	1.28	0.112
Firmicutes	Firmicutes	1	1.07	0.276
Firmicutes	Bacteroidetes	1	1.06	0.014
Bacteroidetes	Bacteroidetes	1	0.90	0.037
Proteobacteria	Bacteroidetes	1	0.40	0.027
Bacteroidetes	Firmicutes	1	0.30	0.038
Euryarchaeota	Firmicutes	1	0.24	0.003

Supplementary Figure 21. Significant co-occurrences are mostly positive. A) Most edges that significantly differed from null model expectations were positive co-occurrences. Each point signifies the expected versus observed co-occurrence among OTUs. B) The table lists the taxonomic composition of the significant edges, with “Sign” signifying a positive (1) or negative (-1) co-occurrence pattern, “Perc. of Edges” signifying the percent of total edges, and “Edges Norm.” signifying the fraction of edges normalized by the sum of nodes associated with those edges.

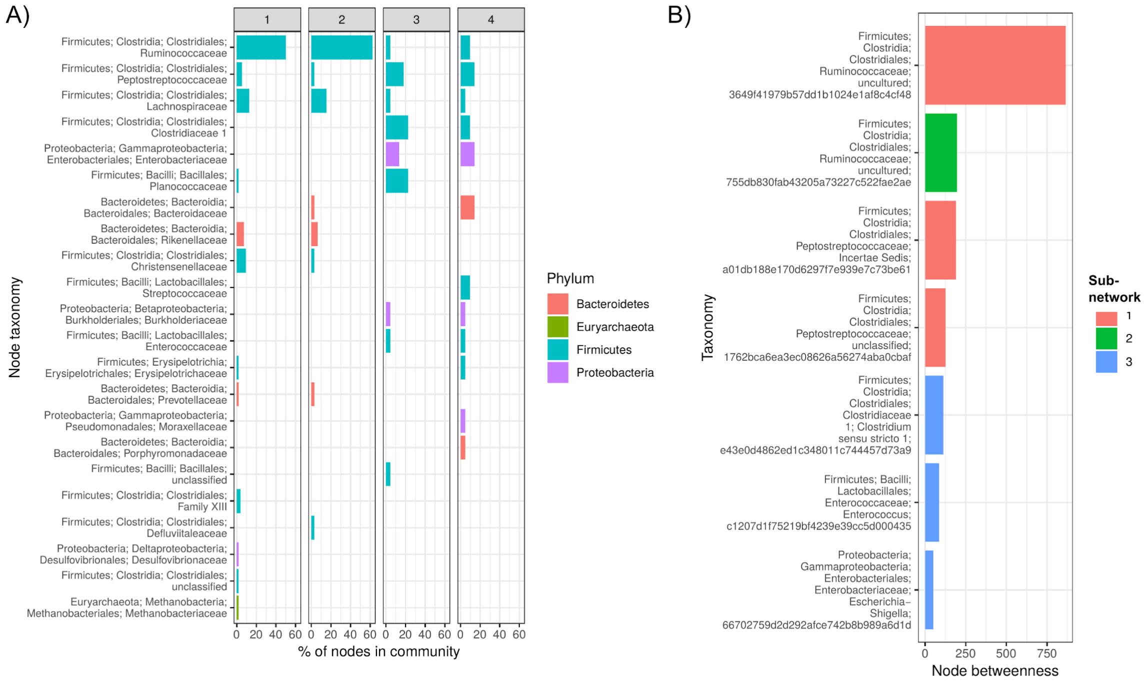

Supplementary Figure 22. A) The bar chart shows the taxonomic composition of the nodes in each sub-network (x-axis plot facet) as defined in Fig. 7, with OTUs grouped at the genus level, and taxon labels listing “Phylum; Class; Order; Family; Genus”. B) The plot shows the centrality betweenness value for all OTUs with a value of >50, and taxon labels are coded as “Phylum; Class; Order; Family; Genus; OTU”. Source data are provided as a Source Data file.
