# Supplementary material for: Host diet and evolutionary history explain different aspects of gut microbiome diversity among vertebrate clades
Source: Nat Commun. 2019 May 16;10:2200. doi: 10.1038/s41467-019-10191-3 (PMC6522487; doi:10.1038/s41467-019-10191-3)
Supplement: Supplementary file 3 — Reporting Summary [file 41467_2019_10191_MOESM3_ESM.pdf]

nature research

Corresponding author(s): Nicholas Youngblut

Last updated by author(s): Apr 11, 2019

Reporting Summary

Nature Research wishes to improve the reproducibility of the work that we publish. This form provides structure for consistency and transparency in reporting. For further information on Nature Research policies, see Authors & Referees and the Editorial Policy Checklist.

Statistics

For all statistical analyses, confirm that the following items are present in the figure legend, table legend, main text, or Methods section.

n/a	Confirmed	
☐	☒	The exact sample size (*n*) for each experimental group/condition, given as a discrete number and unit of measurement
☐	☒	A statement on whether measurements were taken from distinct samples or whether the same sample was measured repeatedly
☐	☒	The statistical test(s) used AND whether they are one- or two-sided<br>*Only common tests should be described solely by name; describe more complex techniques in the Methods section.*
☐	☒	A description of all covariates tested
☐	☒	A description of any assumptions or corrections, such as tests of normality and adjustment for multiple comparisons
☐	☒	A full description of the statistical parameters including central tendency (e.g. means) or other basic estimates (e.g. regression coefficient) AND variation (e.g. standard deviation) or associated estimates of uncertainty (e.g. confidence intervals)
☐	☒	For null hypothesis testing, the test statistic (e.g. *F*, *t*, *r*) with confidence intervals, effect sizes, degrees of freedom and *P* value noted<br>*Give P values as exact values whenever suitable.*
☒	☐	For Bayesian analysis, information on the choice of priors and Markov chain Monte Carlo settings
☒	☐	For hierarchical and complex designs, identification of the appropriate level for tests and full reporting of outcomes
☒	☐	Estimates of effect sizes (e.g. Cohen's *d*, Pearson's *r*), indicating how they were calculated

Our web collection on statistics for biologists contains articles on many of the points above.

Software and code

Policy information about availability of computer code

Data collection	All information on the data collection can be found in the manuscript (e.g., Methods and Supplementary Table 1) along with information at https://github.com/leylabmpi/animal_gut_16S-uni
Data analysis	All code required to reproduce the analysis can be found at https://github.com/leylabmpi/animal_gut_16S-uni

For manuscripts utilizing custom algorithms or software that are central to the research but not yet described in published literature, software must be made available to editors/reviewers. We strongly encourage code deposition in a community repository (e.g. GitHub). See the Nature Research guidelines for submitting code & software for further information.

Data

Policy information about availability of data

All manuscripts must include a data availability statement. This statement should provide the following information, where applicable:

- Accession codes, unique identifiers, or web links for publicly available datasets
- A list of figures that have associated raw data
- A description of any restrictions on data availability

The raw sequence data are available from the European Nucleotide Archive under the study accession number PRJEB29403. Metadata associated with each sample are provided in Supplementary Table 1.

Field-specific reporting

Please select the one below that is the best fit for your research. If you are not sure, read the appropriate sections before making your selection.

☐ Life sciences ☐ Behavioural & social sciences ☒ Ecological, evolutionary & environmental sciences

For a reference copy of the document with all sections, see nature.com/documents/nr-reporting-summary-flat.pdf

Ecological, evolutionary & environmental sciences study design

All studies must disclose on these points even when the disclosure is negative.

Study description	We studied the gut microbiome of wild and captive animals. The major treatments were animal diet and evolutionary history. Other covariates included captivity status (wild vs captive) and sample type (gut contents or feces).
Research sample	The feces (or gut contents) from an individual animal was considered a single sample. We only used one sample per animal species for each hypthesis test.
Sampling strategy	Samples were collected by wildlife biologists. No sample size calculation was performed.
Data collection	Data collection was performed by the wildlife biologists that collected the samples.
Timing and spatial scale	Sampling was conducted from February 2009 and March 2014. Samples originated predominantly from Central Europe (Austria and neighboring countries). However, in order to cover as much vertebrate diversity as possible, many samples were also taken from other countries around the world (see the metadata provided with the manuscript)
Data exclusions	A small subset of samples were excluded due to not enough sampling depth of the microbiome.
Reproducibility	We assessed each question with multiple analyses and compared our results to previous studies in order to assess reproducibility.
Randomization	We utilize a sensitivity method of randomly subsampling one individual sample per species for each hypothesis test, repeating this procedure a total of 100 times, and using the 95% quartile of significance values for each individual subsample to assess overall significance. This allowed us to assess how sensitive our analysis was to intra-species heterogeneity, which would be missed if we had simply used one randomly subsample sample per animal species.
Blinding	No blinding was used. We do not believe it was necessary for our experiment design.

Did the study involve field work? ☒ Yes ☐ No

Field work, collection and transport

Field conditions	Samples were collected from many locations around the world at varying times of the year. See the supplemental metadata provided with the manuscript.
Location	Samples were collected from many locations around the world at varying times of the year. See the supplemental metadata provided with the manuscript.
Access and import/export	Wildlife biologists, who were conducting long-term research on the respective species in its habitat, ensured that sampling guidelines and restrictions were adhered to, where these were applicable. Human DNA samples were taken from a previous study.
Disturbance	No disturbance was caused

Reporting for specific materials, systems and methods

We require information from authors about some types of materials, experimental systems and methods used in many studies. Here, indicate whether each material, system or method listed is relevant to your study. If you are not sure if a list item applies to your research, read the appropriate section before selecting a response.

Materials & experimental systems

n/a	Involved in the study
☒	☐ Antibodies
☒	☐ Eukaryotic cell lines
☒	☐ Palaeontology
☒	☐ Animals and other organisms
☒	☐ Human research participants
☒	☐ Clinical data

Methods

n/a	Involved in the study
☒	☐ ChIP-seq
☒	☐ Flow cytometry
☒	☐ MRI-based neuroimaging
