# Supplementary material for: Host diet and evolutionary history explain different aspects of gut microbiome diversity among vertebrate clades
Source: Nat Commun. 2019 May 16;10:2200. doi: 10.1038/s41467-019-10191-3 (PMC6522487; doi:10.1038/s41467-019-10191-3)
Supplement: Supplementary file 4 — Description of Additional Supplementary Files [file 41467_2019_10191_MOESM4_ESM.pdf]

Description of Additional Supplementary Files

File Name: Supplementary Data 1
Description: Metadata for each sample in the study.

File Name: Supplementary Data 2
Description: Pearson correlation coefficients of LIPA coefficients and 16S rRNA tree branch lengths between OTUs with a significant LIPA coefficient value for at least one host species. Only OTU comparisons with a normalized branch length of < 0.1 and a Pearson coefficient of < 0 are shown. Only OTUs found to have significant LIPA coefficient values in ≥ 1 host species were included in the analysis.

File Name: Supplementary Data 3
Description: Samples from the Earth Microbiome Project that were used to infer biome specificity of taxa (Supplementary Fig. 20).
